# Expression Pattern and Prognostic Value of CTLA-4, CD86, and Tumor-Infiltrating Lymphocytes in Rectal Cancer after Neoadjuvant Chemo(radio)therapy

**DOI:** 10.3390/cancers14225573

**Published:** 2022-11-14

**Authors:** Xin-Ke Yin, Chao Wang, Li-Li Feng, Shao-Mei Bai, Wei-Xing Feng, Neng-Tai Ouyang, Zhong-Hua Chu, Xin-Juan Fan, Qi-Yuan Qin

**Affiliations:** 1 Cellular & Molecular Diagnostics Center, Sun Yat-sen Memorial Hospital, Sun Yat-sen University, Guangzhou 510120, China; 2 Department of Pathology, The Sixth Affiliated Hospital, Sun Yat-sen University, Guangzhou 510655, China; 3 Guangdong Provincial Key Laboratory of Colorectal and Pelvic Floor Diseases, The Sixth Affiliated Hospital, Sun Yat-sen University, Guangzhou 510655, China; 4 Department of Radiation Oncology, The Sixth Affiliated Hospital, Sun Yat-sen University, Guangzhou 510655, China; 5 Department of Gastrointestinal Surgery, Sun Yat-sen Memorial Hospital, Sun Yat-sen University, Guangzhou 510655, China; 6 Department of Colorectal Surgery, The Sixth Affiliated Hospital, Sun Yat-sen University, Guangzhou 510655, China

**Keywords:** immune checkpoint molecules, tumor-infiltrating lymphocytes, neoadjuvant chemo(radio)therapy, immunotherapy, rectal cancer

## Abstract

**Simple Summary:**

Immune checkpoint inhibitors (ICIs) have made an indelible mark on metastatic colorectal cancer patients with microsatellite instability-high (MSI-H) instead of microsatellite stability (MSS) tumors. Clinical studies have explored the introduction of ICIs into treatment for locally advanced rectal cancer patients with MSS; however, the outcomes are still limited. Different studies have provided valuable information for understanding the expression and clinical significance of PD-1 and PD-L1 during neoadjuvant chemoradiotherapy (nCRT). However, the value of CTLA-4 and CD86 after nCRT has not been fully studied. We found that CD86 expression was significantly lower in the nCRT-treated group rather than CTLA-4. Improved overall survival in patients with lower CD86 expression could only be observed in patients with low rather than high t/sCD8^+^ cell densities. Our findings reveal the relationship between the immunosuppressive microenvironment and the CTLA-4/CD86 pathway after nCRT.

**Abstract:**

The synergistic effect of combining immune checkpoint inhibitors (ICIs) with neoadjuvant chemo(radio)therapy (nCRT) in colorectal cancer is still limited. We aimed to understand the impact of nCRT on the tumor microenvironment and to explore favorable immune markers of this combination. Herein, we investigated the expression of cytotoxic T lymphocyte-associated antigen 4 (CTLA-4), CD86, CD4, and CD8 after nCRT and its association with clinicopathological characteristics. Immunostaining of immune-related molecules was performed in 255 surgically resected specimens from rectal cancer patients treated with nCRT. CD4 and CD8 expression on the tumor (tCD4/CD8), stroma (sCD4/CD8), and invasive front (iCD4/CD8) was evaluated. The expression levels of immune-related molecules were significantly lower in the nCRT-treated group, except for CTLA-4 and sCD8. However, patients with higher sCD8^+^ cell density and CTLA-4 expression had better progression-free survival (PFS) and distant metastasis-free survival (DMFS). In addition, higher CD86 expression was associated with poorer overall survival (OS). Higher CTLA-4 expression was associated with higher tCD8^+^ cell density, whereas CD86 expression was correlated with the cell density of t/sCD8. Prognostic analysis confirmed that the relationships between CTLA-4 and DMFS as well as CD86 and OS were significantly correlated in low rather than high CD8^+^ cell density. Further the combination of CD8^+^ cell density and CD86 expression was shown to be an independent prognostic factor of OS, whereas the combination of CTLA-4 was not for DMFS. Together, these results demonstrate significant correlations between CD86 expression and t/sCD8^+^ cell density in rectal cancer after nCRT and could potentially have clinical implications for combining ICIs and nCRT.

## 1. Introduction

Colorectal cancer (CRC) is the third most common cancer worldwide, and patients with metastatic CRC (mCRC) show a poor prognosis, with a five-year survival lower than 20% [1]. Immune checkpoint inhibitors (ICIs) have been approved to treat mCRC patients with mismatch repair-deficient (dMMR) or microsatellite instability-high (MSI-H) tumors due to their long-term durable remission [2]. However, patients with mismatch repair-proficient (pMMR) or microsatellite stability (MSS) tumors constitute approximately 85% of CRC patients and might not benefit from immunotherapy [3,4]. Neoadjuvant chemo(radio)therapy (nCRT/nCT) has been established as the standard treatment for locally advanced rectal cancer (LARC) and plays a crucial role in pathological downstaging and achieving a higher pathologic response rate [5]. Meanwhile, several preclinical data demonstrate that chemotherapy or radiotherapy might generate an increasing neo-antigen repertoire for T cell priming, which could mediate immunostimulatory effects [6,7]. Considering the rising interest in immunotherapeutic strategies after nCRT/nCT, exploring and evaluating the expression of checkpoint-related molecules after neoadjuvant therapies could provide proof of concept for nCRT/nCT in combination with ICIs for treating LARC patients with pMMR/MSS tumors.

ICIs target programmed cell death-1 (PD-1) and cytotoxic T lymphocyte-associated antigen 4 (CTLA-4) on T cells or their ligands, such as programmed cell death 1 ligand-1 (PD-L1) on tumor cells to enhance antitumor T cell activity [8]. In recent studies, PD-L1 expression in rectal cancer (RC) has been shown to increase or decrease during nCRT, with the prognostic values of PD-L1 expression before or after nCRT being dynamic [9,10,11]. Moreover, CD8+ cell densities show different prognostic patterns according to the expression level of PD-L1 [12]. It has been reported that the expression of CTLA-4 is relatively stable after nCRT and has no relationship with OS and DFS in RC [13]. CD86, one of the ligands of CTLA-4, is associated with a higher risk of relapse in patients with chronic myeloid leukemia after discontinuing a tyrosine kinase inhibitor [14]. However, the expression pattern, correlation with tumor-infiltrating lymphocytes, and prognostic value of CTLA-4 and CD86 in RC after nCRT/nCT are yet to be further elucidated.

CTLA-4, also called CD152, is expressed on the surface of CD4^+^ and CD8^+^ T lymphocytes and limits their activation by competing with CD28 for binding to its ligands, CD80/CD86 [15]. While these ligands can both interact with CTLA-4 and CD28, they are only approximately 25% identical in sequence. In recent years, many studies have explored the differences between CD80 and CD86, yet the role of these ligands in T cell regulation is still a paradox. It has been reported that CD80 is the primary ligand mediating CTLA-4 localization, and that CD86 is mainly responsible for CD28 accumulation at the synapse level [16]. On the contrary, in vivo experiments have verified that CTLA-4 can deplete CD80 and CD86 via trogocytosis [17]. However, it is clear that the expression of CD86 is strongly upregulated on dendritic cells by lipopolysaccharide and is generally greater than that of CD80, while CD80 is expressed only after the activation of antigen-presenting cells and temporally after CD86 [18].

Herein, we aimed to detect the expression of CTLA-4, CD86, CD4, and CD8 in surgical specimens from RC patients after nCRT/nCT, and to classify CD4^+^ and CD8^+^ cells into stroma, tumor, and invasive categories according to their location. Subsequently, the relationship between these variables and clinicopathological characteristics was evaluated, including tumor regression grade (TRG) and neoadjuvant therapy methods. We analyzed these variables alongside their clinical outcomes. Furthermore, prognosis focused on CTLA-4 and CD86 expression was analyzed according to the status of CD8^+^ cell density.

## 2. Materials and Methods

### 2.1. Patients and Treatment

Patients with LARC who underwent nCRT following surgical resection at the Sixth Affiliated Hospital of Sun Yat-sen University (SYSU) from 2010 through 2015 were enrolled in the study, and 255 post-nCRT/nCT surgical tissues were collected. All patients signed informed consent, and this study was approved by the Clinical Ethics Review Committee at the Sixth Affiliated Hospital of SYSU. Clinicopathological parameters and follow-up information were retrieved from the Follow-up Database of the Sixth Affiliated Hospital of SYSU. Primary endpoints used in this study included the following: overall survival (OS) was defined as the duration from first diagnosis to death from any cause or last follow-up; progression free survival (PFS) was defined as the duration from total mesorectal excision (TME) surgery to first recurrence or metastasis; distant metastasis free survival (DMFS) was defined as the duration from TME surgery to the first distant relapse (recurrence outside the pelvis).

One hundred and forty-nine patients have been treated with nCRT. For neoadjuvant radiotherapy, the gross tumor volume was treated with 50 Gy in 25 fractions. These patients also received neoadjuvant induction (pre-radiotherapy), as well as concurrent (during treatment) and/or consolidation (post-radiotherapy) chemotherapy according to the LARC guidelines. One hundred and six patients have been treated with nCT. nCT consisted of a fluoropyrimidine-based regimen, involving one of the following: Folinic acid, fluorouracil, and oxaliplatin (FOLFOX); capecitabine and oxaliplatin (CAPOX); capecitabine (Xeloda); folinic acid and fluorouracil (DeGramont). The majority of patients in this cohort also received a fluoropyrimidine-based adjuvant chemotherapy regimen. Total neoadjuvant therapy (TNT) in this study was defined as receiving neoadjuvant radiotherapy, eight or more cycles of nCT, and no adjuvant chemotherapy [19]. The clinical response was assessed four to eight weeks after the completion of chemoradiation according to TME principles.

### 2.2. Grading Standard and Evaluation Method for TRG

The TRG of a primary tumor after preoperative nCRT was evaluated on hematoxylin and eosin-stained slides according to the seventh edition of the American Joint Committee on Cancer-Tumor Regression Grade (AJCC-TRG) [20]: TRG 0—no residual tumor cells, TRG 1—single cell or small group of cells, TRG 2—residual cancer with a desmoplastic response, and TRG 3—minimal evidence of tumor response. Two experienced pathology specialists independently reviewed the tissue sections and evaluated the TRG. When the evaluation results were inconsistent, both discussed and reviewed the sections together and provided the final evaluation. In this study, TRG 0—1 was categorized as the good response group and TRG 2—3 as the poor response group [10,11,13].

### 2.3. Immunohistochemistry (IHC)

The post-treatment surgically resected specimens of all the patients were subjected to an IHC assay. One thousand and twenty sections were deparaffinized in xylene and rehydrated through a series of ethanol solutions, and then the endogenous peroxidase was blocked with a 1% hydrogen peroxide solution. Antigen retrieval was induced with a microwave oven. The sections were incubated with anti-CTLA-4 (1:200, ab19792, Abcam, Cambridge, MA, USA), anti-CD86 (1:200, ab269587, Abcam), anti-CD8 (without dilution, ZA-0508, Zhongshan Golden Bridge Biotechnology, Beijing, China) and anti-CD4 (without dilution, ZA-0519, Zhongshan Golden Bridge Biotechnology) at 4 °C overnight. After washing with PBS, the sections were incubated with secondary antibodies (without dilution, MP-7401/4702, Vectorlabs, Burlingame, CA, USA) at room temperature for 30 min. Immunostaining was performed using a 3,3′-diaminobenzidine (DAB) kit (ZLI-9018, Zhongshan Golden Bridge Biotechnology), which was visualized as a brown-colored precipitate at the antigen site. The sections were then counterstained with hematoxylin, followed by dehydration and mounting. The sections were visualized under a microscope at a 40× magnification (Olympus, DP 27, Tokyo, Japan).

### 2.4. Quantification of the Expression of Immune-Related Molecules

**Figure 1:**
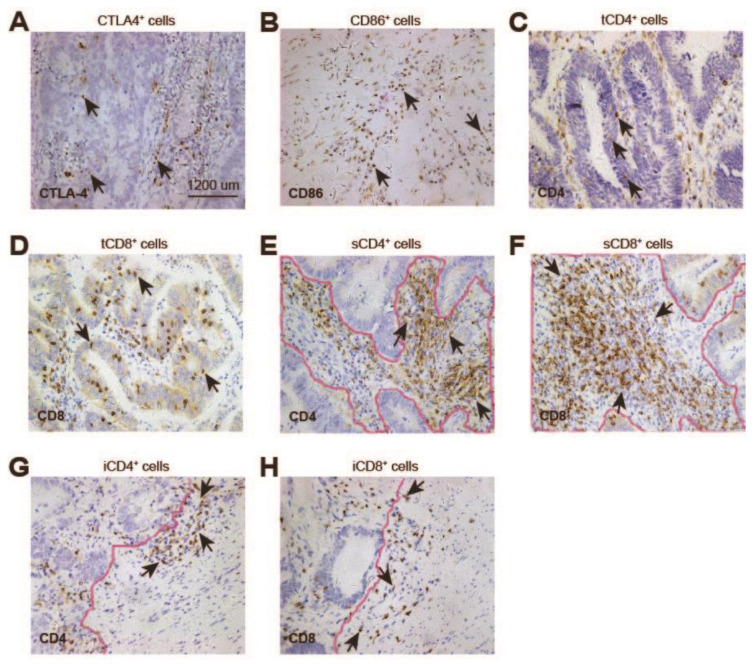
Immunohistochemical staining of immune-related molecules. (**A**) CTLA-4^+^ cells were detected on the stroma and tumor. (**B**) The expression of CD86 was detected on the lymphocytes. (**C**–**H**) CD8 and CD4 positive cells were divided into tCD8^+^/tCD4^+^ cells (lymphocytes on the tumors, (**C**,**D**)), sCD8^+^/sCD4^+^ cells (lymphocytes on the stroma where the curve labeled, (**E**,**F**)), and iCD8^+^/iCD4^+^ cells (lymphocytes on the invasive front, (**G**,**H**)).

The results were assessed by two pathologists who were blinded to the clinical outcomes. CTLA-4 (Figure 1A) and CD86 (Figure 1B) expressed on the immune cells were counted. The expression levels of CD4 and CD8 were evaluated according to the location of the tumors, and then divided into tCD4^+^ and tCD8^+^ cells (lymphocytes on the tumor; Figure 1 C,D), sCD4^+^ and sCD8^+^ cells (lymphocytes on the stroma; Figure 1E,F) and iCD4^+^ and iCD8^+^ cells (lymphocytes on the invasion front; Figure 1G,H). Only s/iCD4^+^ and s/iCD8^+^ cells were counted according to the fibrotic adjacent area in case pathological complete response was evaluated. All the sections were evaluated according to the number of positively stained cells in three randomly selected high-power fields (HPFs) and then averaged. The median counts were set as the thresholds to subdivide the patients.

### 2.5. Statistical Analysis

The association between the CTLA-4^+^, CD86^+^, CD8^+^, and CD4^+^ cells and the patients’ clinicopathological characteristics, therapy method, and treatment response were evaluated using the chi-square test. The Kaplan-Meier curve with the log-rank test were used for survival analysis. Univariate and multivariate analyses were performed using Cox proportional hazard regression via SPSS 25.0 (IBM, Chicago, IL, USA). Forest plots and assumptions for the Cox PH models were performed using the R Survival (v3.4–0) and Survminer (v0.4.9) packages. Differences in CD4^+^ and CD8^+^ cell densities were compared with the paired t test using GraphPad Prism 7.0. All *p* values less than 0.05 were considered statistically significant.

## 3. Results

### 3.1. Patient Characteristics

After applying the inclusion criteria, 255 patients from the Sixth Affiliated Hospital of SYSU cohort were included. The clinicopathological characteristics of the patients are described in Table 1. In brief, there were 178 males (69.8%) and 77 females (30.2%) with a median age of 55 (22–77) years. As for the therapy method, nCRT was given in 149 patients (58.4%), whereas 106 patients (41.6%) received nCT. After treatment, 201 patients were in the N0 stage (78.8% vs. 22.4%) and 124 patients were of T0–T2 stage (48.6% vs. 0%), and 131 patients (51.4%) were categorized as having a good response.

### 3.2. Expression of CTLA-4, CD86, CD4, and CD8 in Rectal Cancer after nCRT

**Figure 2:**
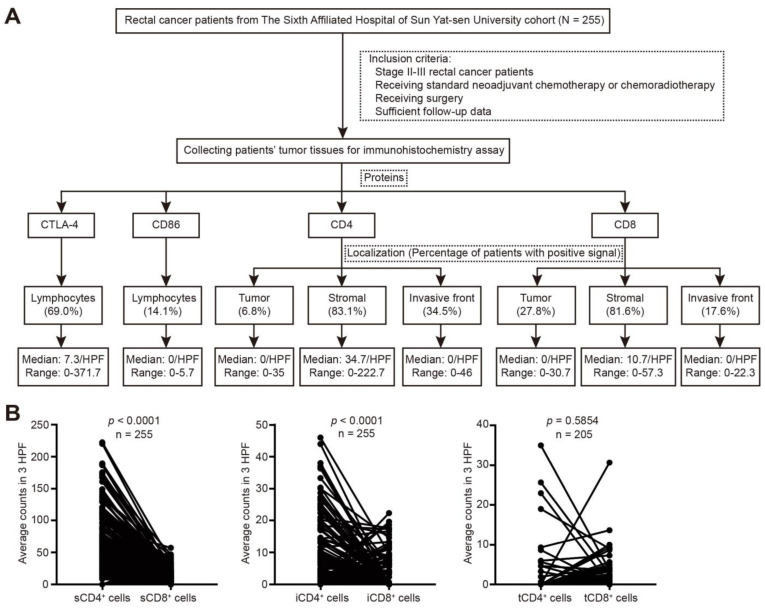
Expression of CTLA-4, CD86, CD4, and CD8 in rectal cancer after nCRT. (**A**) Schematics showing the workflow for detecting and quantifying the expression of immune-related molecules. (**B**) Paired *t* test was performed between s/t/iCD4^+^ and s/t/iCD8^+^ cells.

The expression of immune-related molecules in rectal cancer patients was quantified (Figure 2A). CTLA-4 expression was detected in 176 of the 255 patients (69.0%), and the median counts were 7.3/HPF (range = 0–371.7), while CD86 was only present in 36 of the 255 patients (14.1%) with a median count of 0. CD4 and CD8 were mainly detected in the stroma (34.7/HPF and 10.7/HPF, respectively). The percentage of patients with tCD4^+^ cells was lower than that with tCD8^+^ cells (6.8% vs. 27.8%), and for the invasive front, the ratio of patients with iCD4^+^ cells was higher than that of iCD8^+^ cells (34.5% vs. 17.6%). In addition, CD4 expression was higher than CD8 expression at the stroma and invasive front (*p* < 0.001), whereas the number of tCD4^+^ cells was similar to that of tCD8^+^ cells (*p* = 0.5854; Figure 2B).

### 3.3. Association between Expression of Immune-Related Molecules and Clinicopathologic Characteristics

The relationship between the expression level of immune-related molecules and clinicopathological characteristics was explored. There were no significant differences between the expression of immune-related molecules and ypT or ypN (all *p* > 0.05), expect that high tCD8^+^ cell density was correlated with low ypN grade (*p* = 0.032; Supplementary Table S1). CD86 expression, t/s/iCD4^+^, and t/iCD8^+^ cell densities were significantly lower in the nCRT-treated group (all *p* < 0.05), while CTLA-4 expression and sCD8^+^ cell density were not (both *p* > 0.05; Table 2). Those patients treated with nCRT were more likely to achieve a better response compared to the nCT-treated group (*p* < 0.001; Table 2). In addition, similar associations were observed between the expression of the molecules and TRG categories (Table 2). However, only s/iCD8^+^ cell densities were correlated with TRG categories in patients treated with nCRT (*p* = 0.033 and 0.005, respectively; Supplementary Table S2).

### 3.4. Survival Analysis

**Figure 3:**
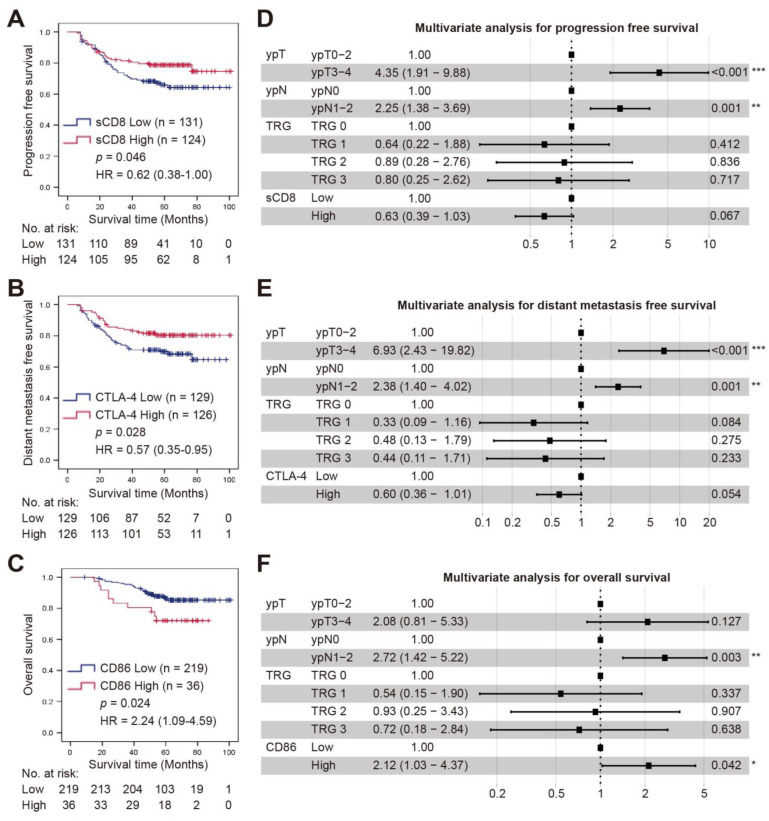
Kaplan-Meier curves and multivariate analysis of survival. (**A**) PFS according to the status of sCD8^+^ cell (low or high). (**B**) DMFS according to CTLA-4^+^ cell density (low or high). (**C**) OS according to CD86 expression (low or high) level. PFS, progression-free survival; DMFS, distant metastasis-free survival; OS, overall survival. (**D**–**F**) Forest plots of PFS (**D**), DMFS (**E**), and OS (**F**) for variables by multivariate Cox analysis. *, *p* < 0.05; **, *p* < 0.01; ***, *p* < 0.001.

The median follow-up duration for PFS, DMFS, and OS was 58.0 months (9–101), 57.0 months (9–101), and 60.0 months (9–101), respectively. Kaplan-Meier curve analysis revealed that compared to the lower expression group, those patients in the higher sCD8^+^ and CTLA-4^+^ cell groups had better PFS and DMFS, respectively (*p* = 0.046 and 0.028; Figure 3A,B), while no correlation was found with OS (*p* = 0.845 and 0.876; Supplementary Figure S1A,B; Supplementary Table S3). However, OS was significantly improved in those patients with lower CD86 expression compared to those with higher CD86 expression (*p* = 0.024; Figure 3C, Supplementary Figure S1C,D; Supplementary Table S3). Furthermore, multivariate analysis showed that sCD8+ cell density and CTLA-4 expression were not the independent prognostic factor of PFS and DMFS, respectively (both *p* > 0.05; Figure 3D,E). CD86 was an independent prognostic factor of OS (HR = 2.12, 95% CI = 1.03–4.37, *p* = 0.042, Figure 3F). Meanwhile, the ypT or ypN stage was an independent prognostic factor of PFS (HR = 4.35, 95% CI = 1.91–9.88, *p* < 0.001; HR = 2.25, 95% CI = 1.38–3.69, *p* = 0.001; respectively), DMFS (HR = 6.93, 95% CI = 2.43–19.82, *p* < 0.001; HR = 2.38, 95% CI = 1.40–4.02, *p* = 0.001; respectively), and OS (HR = 2.72, 95% CI = 1.42–5.22, *p* = 0.003; Figure 3D–F; Supplementary Figure S1E–G). However, the TRG categories were not the independent prognostic factor for OS, PFS, or DMFS (all *p* > 0.05; Figure 3D–F; Supplementary Figure S1E–G).

### 3.5. Combined Status of CD8^+^ Cell Density and CTLA-4/CD86 Expression and Survival

**Figure 4:**
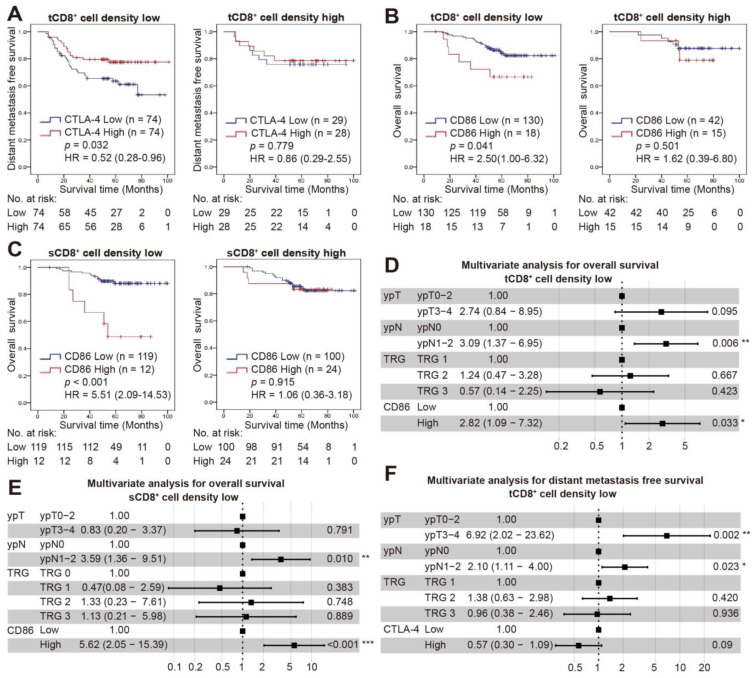
Combined status of CD8^+^ cell density and CTLA-4/CD86 expression with survival. (**A**) Kaplan-Meier curve for DMFS in low or high tCD8+ cell density groups according to CTLA-4 expression. (**B**,**C**) Kaplan-Meier curves for OS in low or high tCD8^+^ (**B**)/sCD8^+^ (**C**) cell density groups according to the CD86 expression. (**D**–**F**) Forest plots of PFS and DMFS for variables by multivariate Cox analysis in low tCD8^+^ (**D**,**F**)/sCD8^+^ (**E**) cell density groups. *, *p* < 0.05; **, *p* < 0.01; ***, *p* < 0.001.

The correlation between CTLA-4/CD86 expression and CD4^+^/CD8^+^ cell density was examined (Table 3). CTLA-4 expression was correlated with the density of tCD8^+^ cells (*p* = 0.003), and the improved DMFS with high CTLA-4 expression was observed in a low, not high tCD8^+^ cell density group (*p* = 0.032 and 0.779, respectively; Figure 4A). In addition, the tCD8^+^/sCD8^+^ cell density was significantly higher in those patients with higher CD86 expression (*p* = 0.013 and 0.019, respectively). CD86 expression was inversely correlated with OS in those patients with a low t/sCD8^+^ cell density (*p* = 0.041 and <0.001, respectively), while it was not observed in those patients with a high t/sCD8^+^ cell density (*p* = 0.501 and 0.915, respectively; Figure 4B,C). Furthermore, besides the ypN stage (HR = 3.09, 95% CI = 1.37–6.95, *p* = 0.006; HR = 3.59, 95% CI = 1.36–9.51, *p* = 0.010), the combination of t/sCD8^+^ cell density and CD86 expression was also an independent prognostic factor of OS (HR = 2.82, 95% CI = 1.09–7.32, *p* = 0.033; HR = 5.62, 95% CI = 2.05–15.39, *p* < 0.001; respectively; Figure 4D,E; Supplementary Table S4; Supplementary Figure S2A,B), while the combination of tCD8+ cell density and CTLA-4 expression was not the independent prognostic factor of DMFS (HR = 0.57, 95% CI = 0.30–1.09, *p* = 0.09; Figure 4F; Supplementary Figure S2C).

## 4. Discussion

Radiotherapy- or chemotherapy-induced tumor cell death can disrupt tumor escape strategies and recruit immune cells to enhance antitumor immunity. Clinical studies have explored the synergy between immunotherapy and conventional therapies [21], as monotherapy no longer meets the actual clinical needs. The effect of combining ICIs with chemo(radio)therapy in RC is still limited. Further understanding of the impact of chemo(radio)therapy on the tumor microenvironment, particularly the CTLA-4 and CD86 signatures, is required to optimize the design of clinical trials. To the best of our knowledge, this is the first study to demonstrate the relationship between CTLA-4, CD86, CD4, and CD8 expression and prognosis after nCRT in RC. Our results showed that the expression of CD86 but not CTLA-4 was significantly lower in the nCRT groups, and that CD86 was an independent prognostic factor of OS. Specifically, those patients with lower CD86 expression could achieve better survival outcomes when their t/sCD8^+^ cell densities were low, and the combined status of t/sCD8^+^ cell density with CD86 expression was an independent prognostic factor of OS.

Usually, flow cytometry is used to identify CD86 expression on B or plasma cells in hematologic malignancies, with a detection rate of 58.0% or 47.5% [22,23]. In solid tumors, CD86 expression can be detected by multiplexed immunofluorescence and is used as the phenotypic biomarker of M1 macrophages, together with Interferon Regulatory Factor 5 (IRF5) [24]. In addition, qRT-PCR, Western blot, and IHC assays can also be used to detect CD86 expression. Herein, CD86 was immunohistochemically detected in 36 of the 255 patients (14.1%), which is similar to nasopharyngeal carcinoma patients (20.0%) [25] and lower in non-small cell lung cancer (NSCLC) patients (53%) [26]. The positive rate of CD86 expression detected by IHC in the present study was slightly lower than in other reports. The levels of soluble immune checkpoint molecules have been reported to decrease during nCRT [27], and CD86 expression showed a different pattern between the nCRT and nCT groups in this study. This indicates that nCRT/nCT might cause a low rate of CD86 expression, and further experiments should be conducted to identify whether the expression of CD86 changes before or after nCRT/nCT.

Studies have also demonstrated that combining ICIs with chemotherapy improves survival not only in patients with metastatic squamous NSCLC [28], but also in patients with stage IB–IIIA NSCLC [29]. The survival advantage is observed only in stage III NSCLC when ICIs are administered after chemoradiotherapy [30,31,32]. For CRC patients with pMMR/MSS tumors, the most favorable time point or therapy methods for an immunotherapy combination remains a great challenge. A subset of tumors might display significant adaptive resistance after post-combined modality therapy, manifested by an active immune microenvironment characterized by increased CD8 infiltration and IFNγ, leading to the compensatory induction of multiple checkpoints, including PD-1, PD-L1, CTLA-4, LAG-3, and IDO, to prevent tumor cell death [33,34]. Furthermore, the expression pattern of the tumor microenvironment shows different changes in different cancer patients after receiving chemo(radio)therapy [35]. Our results showed that the expression of CD86 but not CTLA-4 was significantly higher in the nCT-treated group compared to patients treated with nCRT. Consistent with our study, Teng et al. also verified that CTLA-4 is not associated with the response in rectal cancer [13]. Prognostic analysis showed that CD86 expression was associated with OS, but not with PFS or DMFS. Since a tumor could become more malignant after recurrence or metastasis, the role of CD86 in this stage and the relationship with OS could be further explored. In addition, a previous study shows that the correlation between homologous recombination mutation and prognosis is inconsistent when patients are receiving different types of treatment [36]. Thus, the prognostic value of CD86 could also be explored in patients receiving ICIs, as CD86 plays a vital role in immune response. 

In RC, higher densities of pretreatment CD8^+^ and CD4^+^ cells are associated with a better response and OS [13], while the associations after neoadjuvant treatment have not been thoroughly investigated. We showed that patients with a good response had lower s/iCD8^+^ cell densities only in nCRT-treated group. Moreover, only the expression of sCD8 was associated with PFS after neoadjuvant treatment. Matsutani et al. demonstrated that CD8 expression was significantly higher in the responder group in patients treated with nCT rather than nCRT [37]. However, their 64 patients, whose specimens were available in their study, was much lower than the 255 patients in the present study. In addition, their study did not analyze the relationship between CD8 expression and prognosis according to the tumor’s location, as previous studies have demonstrated the importance of TILs in different regions of the tumor microenvironment for predicting prognosis [38]. Previous studies have demonstrated that improved disease-free survival was only seen in patients with high immune-expressed-PD-L1 [12]. Interestingly, we found that improved DMFS and OS were only observed in patients with low instead of high s/tCD8^+^ cell densities. This suggests that the immunosuppressive microenvironment could affect the function of the CTLA-4/CD86 pathway. In particular, CTLA-4 played a converse role to CD86 in terms of prognosis, with patients with higher CTLA-4 expression having better DMFS. A reduced death rate was observed in NSCLC when considering tumors with CTLA-4 > 20 and Ki67 ≤ 15 [39]. The high CTLA-4 H-score level was correlated with prolonged OS and DFS in extrahepatic bile duct cancer patients plus adjuvant chemoradiotherapy [40]. Although CTLA-4 was not associated with OS and DFS in RC after nCRT [13], these data indicated that the positive prognostic role of CTLA-4 was similar to that of other tumor types. Preclinical models indicate that high-order combined therapy could promote a durable benefit and tumor regression in pancreatic ductal adenocarcinoma [41]. Therefore, in future clinical studies, considering neoadjuvant therapy modalities and CTLA-4/CD86 expression during ICIs, combination therapy might effectively block more immune-evasive tumor mechanisms.

The limitations of this study include its retrospective design at a single institution and its lack of functional experiments. Further in vivo and in vitro studies are needed to verify the role of the CD86 pathway during nCRT. Antibodies and assessment methods for CD86 immunostaining have not been well established, and we used only one antibody to evaluate CD86 staining. In addition, the expression of CD86 was evaluated only in surgical specimens after neoadjuvant therapy for more than four weeks. It would be fascinating to explore whether CD86 expression changes during nCRT. Finally, we only described four immune-related molecules in the present study, and it was hard to build predictive models. Therefore, more immune markers are recommended to establish a comprehensive immune score for ICIs in combination with nCRT.

## 5. Conclusions

In conclusion, tumor immunity was different according to the neoadjuvant therapy method and response, with CD86, CD4, and t/iCD8 expression, but not CTLA-4 and sCD8 expression, being significantly higher in the nCT-treated and poor response groups. However, CTLA-4 and sCD8 expression was correlated with DMFS and PFS, respectively. Furthermore, CTLA-4 expression was related to tCD8^+^ cell density, whereas CD86 expression was associated with t/sCD8^+^ cell density. The relationship between CTLA-4 and DMFS was independent of tCD8^+^ cell density. At the same time, improved OS in patients with lower CD86 expression could only be observed in patients with low t/sCD8^+^ cell density. These findings might have important implications for a possible combined therapy using ICIs after nCRT/nCT in rectal cancer.

## Figures and Tables

**Table 1:** Patient characteristics.

Characteristics	Number of Patients
Gender	
Male	178 (69.8)
Female	77 (30.2)
Age	
≤55	128 (50.2)
>55	127 (49.8)
cT stage	
cT3	203 (79.6)
cT4	52 (20.4)
cN stage	
cN0	57 (22.4)
cN1-2	198 (77.6)
Therapy method	
nCRT	149 (58.4)
nCT	106 (41.6)
ypT	
ypT0-2	124 (48.6)
ypT3-4	131 (51.4)
ypN	
ypN0	201 (78.8)
ypN1-	54 (21.2)
Tumor Regression Grade	
TRG 0	50 (19.6)
TRG 1	81 (31.8)
TRG 2	82 (32.2)
TRG 3	42 (16.5)

TRG, tumor regression grade; nCRT, neoadjuvant chemoradiotherapy; nCT, neoadjuvant chemotherapy.

**Table 2:** Association of immune-related molecules with therapy method and response.

Variables		Therapy Method		*p*	Tumor Regression Grade				*p*
		nCT	nCRT		TRG 0	TRG 1	TRG 2	TRG 3	
CTLA-4^+^ cells	Low	54 (50.9)	75 (50.3)	0.924	26 (52.0)	45 (55.6)	42 (51.2)	16 (38.1)	0.324
	High	52 (49.1)	74 (49.7)		24 (48.0)	36 (44.4)	40 (48.8)	26 (61.9)	
CD86^+^ cells	Low	85 (80.2)	134 (89.9)	0.028 *	47 (94.0)	72 (88.9)	67 (81.7)	33 (78.6)	0.096
	High	21 (19.8)	15 (10.1)		3 (6.0)	9 (11.1)	15 (18.3)	15 (21.4)	
tCD4^+^ cells	Low	85 (88.5)	106 (97.2)	0.014 *	0	79 (97.5)	77 (93.9)	35 (83.3)	0.012 *
	High	11 (11.5)	3 (2.8)		0	2 (2.5)	5 (6.1)	7 (16.7)	
	NA	10	40		50	0	0	0	
sCD4^+^ cells	Low	41 (38.7)	85 (57.0)	0.004 *	31 (62.0)	45 (55.6)	37 (45.1)	13 (31.0)	0.013 *
	High	65 (61.3)	64 (43.0)		19 (38.0)	36 (44.4)	45 (54.9)	29 (69.0)	
iCD4^+^ cells	Low	52 (49.1)	115 (77.2)	0.000 *	44 (88.0)	61 (75.3)	44 (53.7)	18 (42.9)	0.000 *
	High	54 (50.9)	34 (22.8)		6 (12.0)	20 (24.7)	38 (46.3)	24 (57.1)	
tCD8^+^ cells	Low	58 (60.4)	90 (82.6)	0.000 *	0	64 (79.0)	58 (70.7)	26 (61.9)	0.124
	High	38 (39.6)	19 (17.4)		0	17 (21.0)	24 (29.3)	16 (38.1)	
	NA	10	40		50	0	0	0	
sCD8^+^ cells	Low	51 (48.1)	80 (53.7)	0.380	33 (66.0)	40 (49.4)	35 (42.7)	23 (54.8)	0.069
	High	55 (51.9)	69 (46.3)		17 (34.0)	41 (50.6)	47 (57.3)	19 (45.2)	
iCD8^+^ cells	Low	79 (74.5)	131 (87.9)	0.006 *	50 (100.0)	67 (82.7)	61 (74.4)	32 (76.2)	0.002 *
	High	27 (25.5)	18 (12.1)		0 (0.0)	14 (17.3)	21 (25.6)	10 (23.8)	
TRG	TRG 0	10 (9.4)	40 (26.8)	0.000 *	-	-	-	-	-
	TRG 1	24 (22.6)	57 (38.3)		-	-	-	-	
	TRG 2	35 (33.0)	47 (31.5)		-	-	-	-	
	TRG 3	37 (34.9)	5 (3.4)		-	-	-	-	

*, *p* < 0.05, statistically significant; the *p* value of 0.000 indicated *p* < 0.001.

**Table 3:** Correlation between CTLA-4/CD86 expression and CD4^+^/CD8^+^ cells.

Variables		CTLA-4 Expression		*p*	CD86 Expression		*p*
		Low	High		Low	High	
tCD4^+^ cells	Low	98 (95.1)	93 (91.2)	0.260	162 (94.2)	29 (87.9)	0.188
	High	5 (4.9)	9 (8.8)		10 (5.8)	4 (12.1)	
sCD4^+^ cells	Low	66 (51.2)	60 (47.6)	0.571	109 (49.8)	17 (47.2)	0.777
	High	63 (48.8)	66 (52.4)		110 (50.2)	19 (52.8)	
iCD4^+^ cells	Low	90 (69.8)	77 (61.1)	0.146	146 (66.7)	21 (58.3)	0.330
	High	39 (30.2)	49 (38.9)		73 (33.3)	15 (41.7)	
tCD8^+^ cells	Low	84 (81.6)	64 (62.7)	0.003 *	130 (75.6)	18 (54.5)	0.013 *
	High	19 (18.4)	38 (37.3)		42 (24.4)	15 (45.5)	
sCD8^+^ cells	Low	68 (52.7)	63 (50.0)	0.665	119 (54.3)	12 (33.3)	0.019 *
	High	61 (47.3)	63 (50.0)		100 (45.7)	24 (66.7)	
iCD8^+^ cells	Low	108 (83.7)	102 (81.0)	0.562	184 (84.0)	26 (72.2)	0.085
	High	21 (16.3)	24 (19.0)		35 (16.0)	10 (27.8)	

*, *p* < 0.05, statistically significant.

## Data Availability

The data that support the findings of this study are available from the corresponding author upon reasonable request.

## Supplementary Materials

The following supporting information can be downloaded at: https://www.mdpi.com/article/10.3390/cancers14225573/s1, Supplementary Table S1, Correlation between the expression of immune-related molecules and clinicopathologic characteristics; Supplementary Table S2, Association of immune-related molecules with TRG categories in nCT- and nCRT-treated groups; Supplementary Table S3, Univariate analysis for survival; Supplementary Table S4, Multivariate analysis for overall survival for patients with TRG 1-3 and low sCD8^+^ cell density; Supplementary Figure S1, Kaplan-Meier curves and multivariate analysis of survival; and Supplementary Figure S2, The assumption of Cox PH model test.
