# Supplementary material for: Expression Pattern and Prognostic Value of CTLA-4, CD86, and Tumor-Infiltrating Lymphocytes in Rectal Cancer after Neoadjuvant Chemo(radio)therapy
Source: Cancers (Basel). 2022 Nov 14;14(22):5573. doi: 10.3390/cancers14225573 (PMC9688334; doi:10.3390/cancers14225573)
Supplement: Supplementary file 1 [file cancers-14-05573-s001.zip › cancers-1918799-supplementary.pdf]

Expression pattern and prognostic value of CTLA-4, CD86, and tumor-infiltrating lymphocytes in rectal cancer after neoadjuvant chemo(radio)therapy

<u>Inventory of supplementary data</u>

1. Supplementary Tables……………………… …………………………...Page 2-5

2. Supplementary Figure and legend……………………………………......Page 6

Supplementary Table S1. Correlation between the expression of immune-related molecules and clinicopathologic characteristics.

Variables		CTLA-4⁺ cells		p	tCD4⁺ cells		p	sCD4⁺ cells		p	iCD4⁺ cells		p
		Low	High		Low	High		Low	High		Low	High	
Age	≤55	67 (51.9)	61 (48.4)	0.573	92 (48.2)	8 (57.1)	0.517	64 (50.8)	64 (49.6)	0.850	86 (51.5)	42 (47.7)	0.567
	> 55	62 (48.1)	65 (51.6)		99 (51.8)	6 (42.9)		62 (49.2)	65 (50.4)		81 (48.5)	46 (52.3)	
Sex	Male	92 (71.3)	86 (68.3)	0.594	136 (71.2)	9 (63.4)	0.583	91 (72.2)	87 (67.4)	0.406	123 (73.7)	55 (62.5)	0.065
	Female	37 (28.7)	40 (31.7)		55 (28.8)	5 (35.7)		35 (27.8)	42 (32.6)		44 (26.3)	33 (37.5)	
cT stage	cT3	101 (78.3)	102 (81.0)	0.598	150 (78.5)	12 (85.7)	0.524	93 (73.8)	110 (85.3)	0.023 *	127 (76.0)	76 (86.4)	0.052
	cT4	28 (21.7)	24 (19.0)		41 (21.5)	2 (14.3)		33 (26.2)	19 (14.7)		40 (24.0)	12 (13.6)	
cN stage	cN0	23 (17.8)	34 (27.0)	0.079	40 (20.9)	3 (21.4)	0.966	36 (28.6)	21 (16.3)	0.018 *	39 (23.4)	18 (20.5)	0.597
	cN1–2	106 (82.2)	92 (73.0)		151 (79.1)	11 (78.6)		90 (71.4)	108 (83.7)		128 (76.6)	70 (79.5)	
ypT	ypT0–2	62 (48.1)	62 (49.2)	0.855	67 (35.1)	7 (50.0)	0.262	63 (50.0)	61 (47.3)	0.665	87 (52.1)	37 (42.0)	0.127
	ypT3–4	67 (51.9)	64 (50.8)		124 (64.9)	7 (50.0)		63 (50.0)	68 (52.7)		80 (47.9)	51 (58.0)	
ypN	ypN0	97 (75.2)	104 (82.5)	0.151	145 (75.9)	10 (71.4)	0.706	101 (80.2)	100 (77.5)	0.606	135 (80.8)	66 (75.0)	0.278
	ypN1–2	32 (24.8)	22 (17.5)		46 (24.1)	4 (28.6)		25 (19.8)	29 (22.5)		32 (19.2)	22 (25.0)	

*, $p < 0.05$, statistically significant; The p value of 0.000 indicated $p < 0.001$.

Supplementary Table S1. Correlation between the expression of immune-related molecules and clinicopathologic characteristics (continued).

Variables		CD86⁺ cells (%)			tCD8⁺ cells (%)			sCD8⁺ cells (%)			iCD8⁺ cells (%)		
		**Low**	**High**	***p***	**Low**	**High**	***p***	**Low**	**High**	***p***	**Low**	**High**	***p***
Age	≤ 55	109 (49.8)	19 (52.8)	0.738	74 (50.0)	26 (45.6)	0.574	68 (51.9)	60 (48.4)	0.574	110 (52.4)	18 (40.0)	0.132
	> 55	110 (50.2)	17 (47.2)		74 (50.0)	31 (54.4)		63 (48.1)	64 (51.6)		100 (47.6)	27 (60.0)	
Sex	Male	152 (69.4)	26 (72.2)	0.733	104 (70.3)	41 (71.9)	0.815	91 (69.5)	87 (70.2)	0.904	149 (71.0)	29 (64.4)	0.388
	Female	67 (30.6)	10 (27.8)		44 (29.7)	16 (28.1)		40 (30.5)	37 (29.8)		61 (29.0)	16 (35.6)	
cT stage	cT3	173 (79.0)	30 (83.3)	0.549	112 (75.7)	50 (87.7)	0.058	100 (76.3)	103 (83.1)	0.183	162 (77.1)	41 (91.1)	0.035 *
	cT4	46 (21.0)	6 (16.7)		36 (24.3)	7 (12.3)		31 (23.7)	21 (16.9)		48 (22.9)	4 (8.9)	
cN stage	cN0	47 (21.5)	10 (27.8)	0.399	35 (23.6)	8 (14.0)	0.130	34 (26.0)	23 (18.5)	0.156	45 (21.4)	12 (26.7)	0.444
	cN1–2	172 (78.5)	26 (72.2)		113 (76.4)	49 (86.0)		97 (74.0)	101 (81.5)		165 (78.6)	33 (73.3)	
ypT	ypT0–2	109 (49.8)	15 (41.7)	0.367	50 (33.8)	24 (42.1)	0.266	64 (48.9)	60 (48.4)	0.940	107 (51.0)	17 (37.8)	0.109
	ypT3–4	110 (50.2)	21 (58.3)		98 (66.2)	33 (57.9)		67 (51.1)	64 (51.6)		103 (49.0)	28 (62.2)	
ypN	ypN0	177 (80.8)	24 (66.7)	0.054	106 (71.6)	49 (86.0)	0.032 *	103 (78.6)	98 (79.0)	0.937	165 (78.6)	36 (80.0)	0.831
	ypN1–2	42 (19.2)	12 (33.3)		42 (28.4)	8 (14.0)		28 (21.4)	26 (21.0)		45 (21.4)	9 (20.0)	

*, $p < 0.05$, statistically significant; The p value of 0.000 indicated $p < 0.001$.

Supplementary Table S2. Association of immune-related molecules with TRG categories in nCT- and nCRT-treated groups

Variables		TRG of nCT-treated group					TRG of nCRT-treated group				
		TRG 0	TRG 1	TRG 2	TRG 3	*p*	TRG 0	TRG 1	TRG 2	TRG 3	*p*
CTLA-4+ cells	Low	7 (70.0)	14 (58.3)	19 (54.3)	13 (35.1)	0.122	19 (47.5)	31 (54.4)	23 (48.9)	2 (40.0)	0.861
	High	3 (30.0)	10 (41.7)	16 (45.7)	24 (64.9)		21 (52.5)	26 (45.6)	24 (51.1)	3 (60.0)	
$CD86^+$ cells	Low	10 (100.0)	19 (79.2)	27 (77.1)	29 (78.4)	0.429	37 (92.5)	53 (93.0)	40 (85.1)	4 (80.0)	0.452
	High	0 (0.0)	5 (20.8)	8 (22.9)	8 (21.6)		3 (7.5)	4 (7.0)	7 (14.9)	1 (20.0)	
$tCD4^+$ cells	Low	NA	23 (95.8)	32 (91.4)	30 (81.1)	0.167	NA	56 (98.2)	45 (95.7)	5 (100.0)	0.687
	High	NA	1 (4.2)	3 (8.6)	7 (18.9)		NA	1 (1.8)	2 (4.3)	0 (0.0)	
$sCD4^+$ cells	Low	5 (50.0)	13 (54.2)	21 (60.0)	14 (37.8)	0.289	24 (60.0)	31 (54.4)	18 (38.3)	2 (40.0)	0.187
	High	5 (50.0)	11 (45.8)	14 (40.0)	23 (62.2)		16 (40.0)	26 (45.6)	29 (61.7)	3 (60.0)	
$iCD4^+$ cells	Low	10 (100.0)	24 (100.0)	35 (100.0)	37 (100.0)	NA	40 (100.0)	56 (98.2)	47 (100.0)	5 (100.0)	0.654
	High	0 (0.0)	0 (0.0)	0 (0.0)	0 (0.0)		0 (0.0)	1 (1.8)	0 (0.0)	0 (0.0)	
$tCD8^+$ cells	Low	NA	13 (54.2)	23 (65.7)	22 (59.5)	0.665	NA	51 (89.5)	35 (74.5)	4 (80.0)	0.132
	High	NA	11 (45.8)	12 (34.3)	15 (40.5)		NA	6 (10.5)	12 (25.5)	1 (20.0)	
$sCD8^+$ cells	Low	6 (60.0)	9 (37.5)	17 (48.6)	22 (59.5)	0.359	27 (67.5)	29 (50.9)	17 (36.2)	2 (40.0)	0.033 *
	High	4 (40.0)	15 (62.5)	18 (51.4)	15 (40.5)		13 (32.5)	28 (49.1)	30 (63.8)	3 (60.0)	
$iCD8^+$ cells	Low	10 (100.0)	16 (66.7)	24 (68.6)	29 (78.4)	0.162	40 (100.0)	51 (89.5)	37 (78.7)	3 (60.0)	0.005 *
	High	0 (0.0)	8 (33.3)	11 (31.4)	8 (21.6)		0 (0.0)	6 (10.5)	10 (21.3)	2 (40.0)	

TRG, tumor regression grade; nCRT, neoadjuvant chemoradiotherapy; nCT, neoadjuvant chemotherapy.

*, $p < 0.05$, statistically significant; The *p* value of 0.000 indicated $p < 0.001$.

Supplementary Table S3. Univariate analysis for survival.

Variables	PFS	DMFS	OS
	p	***p***	***p***
Age (> 55 vs ≤55)	0.833	0.948	0.272
Gender (Female vs Male)	0.976	0.981	0.501
cT stage (cT4 vs cT3)	0.182	0.138	0.156
cN stage (cN1-cN2 vs cN0)	0.250	0.312	0.407
Therapy method (nCT vs nCRT)	0.944	0.675	0.617
ypT (ypT3-ypT4 vs ypT0-ypT2)	0.000 *	0.000 *	0.009 *
ypN (ypN1-ypN2 vs ypN0)	0.000 *	0.000 *	0.000 *
TRG (TRG 3 vs TRG 2 vs TRG 1 vs TRG 0)	0.001 *	0.002 *	0.060
CTLA-4^+ cells (High vs Low)	0.145	0.031 *	0.876
$CD86^+$ cells (High vs Low)	0.757	0.942	0.028 *
$tCD4^+$ cells (High vs Low)	0.503	0.679	0.791
$sCD4^+$ cells (High vs Low)	0.413	0.512	0.308
$iCD4^+$ cells (High vs Low)	0.346	0.366	0.987
$tCD8^+$ cells (High vs Low)	0.330	0.298	0.452
$sCD8^+$ cells (High vs Low)	0.049 *	0.053	0.845
$iCD8^+$ cells (High vs Low)	0.568	0.830	0.922

PFS, progression free survival; DMFS, distant metastasis free survival; OS, overall survival.

*, $p < 0.05$, statistically significant; The *p* value of 0.000 indicated $p < 0.001$.

Supplementary Table S4. Multivariate analysis for overall survival for patients with TRG 1-3 and low $sCD8^+$ cell density.

Variables		HR	95% CI	*p*
ypT	ypT0-2	1.00		
	ypT3-4	0.80	0.19-3.33	0.764
ypN	ypN0	1.00		
	ypN1-2	3.76	1.27-11.13	0.017 *
TRG	TRG 1	1		
	TRG 2	3.00	0.73-12.23	0.127
	TRG 3	2.42	0.53-11.08	0.256
sCD8_low/CD86	Low	1		
	High	5.65	1.84-17.37	0.003 *

*, $p < 0.05$, statistically significant.

Supplementary Figure S1

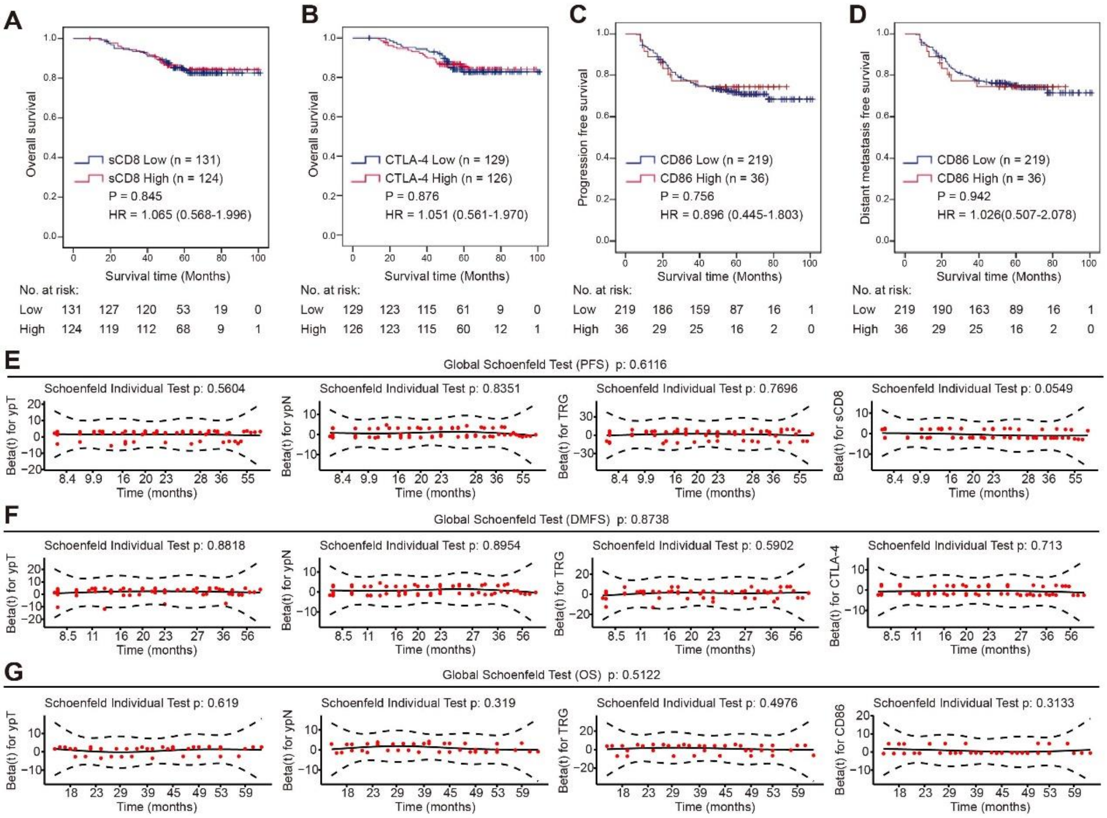

Supplementary Figure S1. Kaplan-Meier curves and multivariate analysis of survival. Kaplan-Meier curves of OS according to the expression of sCD8 (A) and CTLA-4 (B). Kaplan-Meier curves of PFS (C) and DMFS (D) according to CD86 expression. Tested the assumptions of Cox PH model for PFS (E), DMFS (F), and OS (G). PFS, progression free survival; DMFS, distant metastasis free survival; OS, overall survival.

Supplementary Figure S2

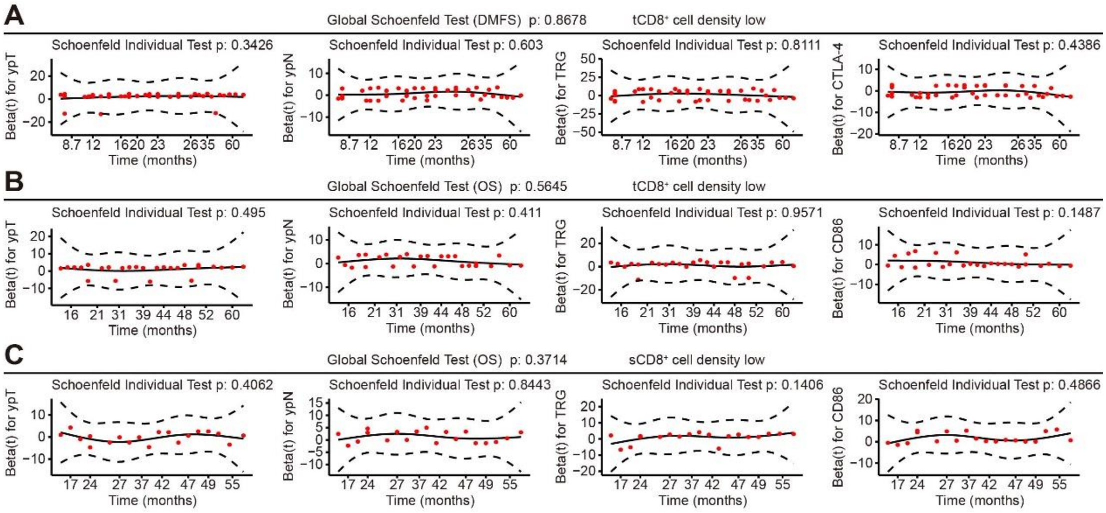

Supplementary Figure S2. The assumption of Cox PH model test. Tested the assumptions of Cox PH model for DMFS and OS according to $tCD8^+$ (A,B) and $sCD8^+$ (C) cell density low.
